# The promising effects of BMP2 transfected mesenchymal stem cells on human osteosarcoma

**DOI:** 10.3906/biy-2101-50

**Published:** 2021-06-23

**Authors:** Ahmet Sinan SARI, Emre DEMİRÇAY, Ahmet ÖZTÜRK, Ayşen TERZİ, Erdal KARAÖZ

**Affiliations:** 1 Department of Orthopedics and Traumatology, Faculty of Medicine, Başkent University, Ankara Training and Research Hospital, Ankara Turkey; 2 Department of Orthopedics and Traumatology, Faculty of Medicine, Başkent University, Istanbul Training and Research Hospital, İstanbul Turkey; 3 Stem Cell and Gene Therapy Research and Application Center, Kocaeli Turkey; 4 Department of Pathology, Faculty of Medicine, Başkent University, Ankara Training and Research Hospital, Ankara Turkey; 5 Istinye University, School of Medicine, Department of Histology and Embryology, İstanbul Turkey; 6 Istinye University, Center for Stem Cell and Tissue Engineering Research and Practice, İstanbul Turkey; 7 Liv Hospital, Center for Regenerative Medicine and Stem Cell Manufacturing (LivMedCell), İstanbul Turkey

**Keywords:** Intraperitoneal, BMP2, tumor, lung metastasis, osteogenic differentiation, targeting

## Abstract

Selective targeting of transfected mesenchymal stem cells (MSCs) carrying specific antioncogenes to the tumor was suggested as a treatment option. Bone morphogenetic protein-2 (BMP2) was shown to inhibit the proliferation and aggressiveness of osteosarcoma (OS) cells. Here, we aimed to assess the homing efficiency of intraperitoneally administered hMSCs transfected with *BMP2* to the tumoral site and their effects on OS using an orthotopic xenograft murine model. Orthotopic xenograft murine model of OS in six-week-old female NOD/SCID mice using 143B cells was established. hMSCs transfected with *BMP2* (BMP2^+^hMSC) were used. In vivo experiments performed on four groups of mice that received no treatment, or intraperitoneally administered BMP2, hMSCs, and BMP2^+^hMSCs. Histopathological and immunohistochemical studies were used to evaluate the pathological identification and to assess the dimensions and necrotic foci of the tumor, the features of lung metastases, and immunostaining against p27, Ki-67, and caspase-3 antibodies. The osteogenic differentiation markers BMP2, BMP4, COL1A1, OPN, OCN and PF4 evaluated using RT-PCR. The tumor dimensions in the hMSCs group were significantly higher than those of the remaining groups (p < 0.01). The number of metastatic foci in the BMP2^+^hMSCs group was significantly lower than those of the other groups (p < 0.01). The current results showed that the intraperitoneal route could be efficiently used for targeting hMSCs to the tumoral tissues for effective BMP2 delivery. In this study, the effects of BMP2 transfected hMSCs on human OS and metastasis were promising for achieving osteogenic differentiation and reduced metastatic process.

## 1. Introduction

Osteosarcoma (OS), the most common sarcoma of bone, which is characterized by osteoid and bone production by malignant spindle cells, usually occurs at the metaphyseal regions of the long bones with a high osteoblastic activity and populated with rapidly dividing mesenchymal cells and preosteoblasts (Aydin et al., 2015; Fan et al., 2020). Mesenchymal stem cells (MSCs) capable of self-renewal, proliferation, and differentiation give rise to osteoblasts within a sophisticated network of genes and factors that play distinct roles in complex signaling pathways. MSCs can secrete various paracrine factors interacting with other cells, and they can selectively target specific areas in the body. These characteristics seem very promising in the treatment of disorders like stroke, myocardial infarction, and various malignant tumors (Luo et al., 2008).

The osteogenic differentiation pathway is coordinated by various growth and differentiation factors (Rutkovskiy et al., 2016). Bone morphogenetic proteins (BMPs), Runx2, collagen type 1 alpha 1 (COL1A1), osteocalcin (OCN), and osteopontin (OPN) are among the factors that pave the road from proliferation to differentiation of MSC towards an osteocyte (Luo et al., 2008; Rutkovskiy et al., 2016). The invariable presence of an osteoid matrix in tumor tissue is evidence for terminal differentiation defect of osteoblasts in the pathogenesis of OS, which occurs when osteoblasts survive despite any disturbance during the differentiation phase of osteogenesis (Luo et al., 2008). Although classified as a rare tumor type, the five-year survival rate of OS is 20% in adolescents, and mortality is frequently due to lung metastasis (Fan et al., 2020).

Target-selective treatments for increasing survival and decreasing side effects of conventional therapies have been the focus of recent studies. Advances in genetic engineering have enabled the potential use of stem cell-based therapeutics as a promising option in OS (Chindamo et al., 2020). The specific features inherent to human MSCs (hMSCs) encouraged researchers to investigate ways of their therapeutic applications. The selective targeting of transfected MSCs with specific antioncogenic factors to the tumor site was suggested to be an effective treatment option (NguyenThai et al., 2015). 

As members of the transforming growth factor-β (TGF-β) protein superfamily, BMPs participate in a variety of biological processes, like angiogenesis and tissue fibrosis. With such diversity of functions, BMPs have gained immediate interest for therapeutic use in tissue engineering and biomedical regenerative therapies. On top of all, there are some studies that investigated the use of BMP2 in the treatment of oncologic conditions (Xiong et al., 2018). For example, exogenous BMP2 administration had been shown to inhibit the proliferation and aggressive properties of human colorectal cancer cells (Zhang et al., 2014). On the contrary, BMP2 has recently been shown to promote the growth of cells of the 143B OS line and enhance their mobility and invasiveness, suggestively through Wnt/beta-catenin signaling pathway (Tian et al., 2019). 

The interactions among OS and hMSCs in the presence of BMP2 were explored by the degree of differentiation of tumoral cells using osteogenic markers like COL1A1, OPN, and OCN that are sequentially expressed matching the temporal pattern of osteoblastic differentiation (Edgar et al., 2007). While COL1A1 appears as an early differentiation marker, OPN peaks in dual-mode during the proliferation and end-proliferation stages, and OCN emerges as a late marker of differentiation (Huang et al., 2007). OPN, which is a late marker of osteogenic differentiation and a phosphoprotein involved in the proliferative, migrative, and adhesive properties of hMSCs in a bidirectional way, is expressed in significantly lower amounts in OS cells compared to differentiated mature osteoblasts (Si et al., 2020). The expression of OCN elevates with the transformation of preosteoblasts to fully differentiated osteoblasts (Miron and Zhang, 2012). Some cytokines like BMP2 and BMP4 modulate the osteoblastic differentiation process with a temporal expression pattern, i.e. earlier, and gradually diminishing expression of BMP4 and later and more constant expression of BMP2 (Edgar et al., 2007; Huang et al., 2007). The expression of BMP4 was shown to induce MSCs towards osteogenic lineage (Wright et al., 2002). In the osteoblast-differentiated MSC cell line Ost-B10, the osteoblastic differentiation had been associated with increased levels of platelet factor 4 (PF4), which is a factor in blood coagulation and modulator of cellular migration (Mishima et al., 2010).

The only conclusion that could be safely drawn from the current literature is the unavailability of consistency among results of studies on the efficiency and the safety of using BMP2 and hMSCs together or separately in OS treatment. Overall, the effect of MSCs from different origins that were transfected with various BMP types on OS has been investigated in a number of experimental studies in animals, while there are no such studies in human OS tissue to the best of our knowledge (Thawani et al., 2010; NguyanThai et al., 2015; Qiao et al., 2015; Tian et al., 2019). In this study, we aimed to assess the homing efficiency of intraperitoneally administered hMSCs transfected with *BMP2* and their effects on human OS and lung metastasis in vitro and in vivo experiments using an orthotopic xenograft murine model. 

## 2. Materials and methods

### 2.1. Study design

This study was conducted in the Department of Orthopedics, Faculty of Medicine, Başkent University, designed to consist of in vitro and in vivo experiments. All animal experiments complied with the Animal Research: Reporting of in vivo Experiments version 2.0 (The ARRIVE guidelines 2.0) and have been carried out according to the Institutional Ethics Committee Regulations and National Institutes of Health guide for the care and use of Laboratory Animals (NIH Publications No.9-8023, revised 1978).

### 2.2. Cell lines and experimental animals

The animals were housed at 20–22 ºC with 12 h light/dark cycle. The human OS cell line, 143B, was purchased from the American Type Culture Collection (ATCC; CRL-8303). The human bone marrow MSC line defined previously (Karaöz et al., 2011) used from institutional cell bank. Cell culture reagents were purchased from Thermo Fisher Scientific (Paisley, UK) unless noted otherwise.

### 2.3. Cell cultures and hMSCs labeling with green fluorescent protein (GFP)

The culture conditions for 143B and hMSCs lines were adjusted according to previous works (Karaöz et al., 2011; Garimella et al., 2017). *Mycoplasma* contamination was tested using a PCR-based kit (EZ-PCR *Mycoplasma* Detection Kit, #20-700-20, Biological Industries, Beit-Haemek, Israel), and subculturing was performed when 70%–80% confluency was achieved. The hMSCs at passage 4 were labeled with GFP (designated as hMSCs for simplification), as described previously (Adas et al., 2016). For the cell labeling, pAcGFP-N1vector (Clontech, Mountain View, CA, USA) was transfected using Neon Transfection System (Invitrogen, Life Technologies, Carlsbad, CA, USA) with the previously optimized settings of 990 V, 20 ms and 2 pulses.

### 2.4. hMSCs transfection with GFP and BMP2

*Escherichia Coli (E. coli)* (NEB 5-alpha, #C2987I, New England BioLabs, Frankfurt, Germany, GeneBank/EMBL accession #Y14837) was propagated in LB supplied with 15 g/L agar, 10 g/L Tryptone, 5 g/L yeast extract, and 0.5 g NaCl at 37 ºC. Human BMP2 gene (NM_001200.2) was purchased on pUC57 (2710 bp, #SD1176, GenScript, Piscataway, Township, NJ, USA). After the subcloning, the vector with *BMP2* gene was purified with Fast Plasmid Mini Kit (5PRIME, Hamburg, Germany). The amplification of the BMP2 fragment with proper restriction enzyme sites was performed in PCR Thermal Cycler Dice (Takara, Tokyo, Japan) using Phusion High-Fidelity DNA polymerase (#F530S, Thermo Fisher Scientific, Leon-Rot, Germany) using the primer pairs F:5’-GGATCCATGGTAGCCGGGAC-3’ and R:5’-GGATCCTAGCGACACCCACAACC at Tm = 58 ºC. The amplified fragments were purified from the PCR reactionby PCR Agarose Gel Extract Mini Kit (5PRIME). BamHI restriction enzyme (#ER0051, Fermentas, Thermo Fisher Scientific, Vilnius, Lithuania) was used to digest both the amplified *BMP2* fragment and the expression vector (pFLAG-CMV™-3, #E6783, Sigma-Aldrich, St. Louis, MO, USA). T4 DNA ligase (#EL0014, Thermo Fisher Scientific, Vilnius, Lithuania) was used to ligate the *BMP2* fragment adjacent to the signal peptide sequence of the vector. *E. coli* was transfected with the constructed plasmid and allowed to propagate in LB under selective conditions against Kanamycin (35 mg/dL, Merck, Darmstadt, Germany). After purification of BMP2 plasmid from *E. coli* cells, PicoDrop spectrophotometer (Picodrop Limited, Saffron, Walden, UK) at 260 nm wavelength was used for estimating the concentration of the product. The size of the *BMP2* fragment on pFLAG-CMV-3-BMP2 was verified by PCR. Electroporation by Neon Transfection System was used for the cotransfection of hMSCs at passage 4 with 0.7 µg pAcGFP-N1 and 4 µg pFLAG-CMV-3-BMP2 (6:1, mol/mol), and under selective condition of 0.2 mM G418 (Thermo Fisher Scientific, Gibco), hMSCs expressing both *GFP* and *BMP2* were obtained. For simplification, this cell line was designated as BMP^+^hMSCs.

### 2.5. Coculture of 143B and BMP2+hMSCs cell lines 

143B cells were cultured on a 6-well plate (3 × 10^4^ cells/well) for 24 h. hMSCs or BMP2^+^hMSCs (both 3 × 10^4^ cells/well) cultured for 24 h on 1 µm permeable wells (#353102, Falcon, BD Biosciences, Bedford, MA, USA) were seeded on permeable insert (3 µm, #353091, Falcon, BD Biosciences) for coculturing for 48 h. 

### 2.6. Detection of BMP2 protein levels in cultures using ELISA 

The BMP2 levels in hMSCs and BMP2^+^hMSCs cultures, and 143B+hMSCs and 143B+BMP2^+^hMSCs cocultures were measured (×3 for each sample) in the 4th and 14th culture days using human BMP2 ELISA kit (#EK0311, Boster, Pleasanton, CA, USA) according to the manufacturer’s instructions. After 2 days of culture in DMEM, 500 µL of culture medium was used to analyze the level of secreted BMP2 level.

### 2.7. Orthotopic xenograft murine model of human OS

The Institutional Animal Care and Use Committee and Ethical Committee of Kobay DHL A.Ş. approved the study protocol (Approval date 05.03.2013 and approval number 61). Six-week-old female nonobese diabetic/severe combined immunodeficiency (NOD/SCID) mice (Jackson Laboratory, Bar Harbor, ME, USA) used for orthotopic xenograft transplantation were housed under previously defined conditions. The left proximal tibia of all mice was injected with 1 × 10^6^ 143B cells suspended in 50 µL (2 × 10^7^ cells/mL) on Day 0, in the phosphate-buffered saline (PBS) (SIGMA, Cat No: 806552) as described (Luu et al., 2005; Luo et al., 2008). 

The mice were divided into four groups. Group 1 (n = 12) (control) received no additional treatment. Group 2 (n = 14), group 3 (n=12), and group 4 (n=12) received intraperitoneal administration of 100 mcg/day BMP2 protein (catalog number: 34-8507-85) (14th and 21stdays), 1 × 10^6^/75 µL hMSCs (14thday), and 1 × 10^6^/75 µL BMP2^+^hMSCs (14th day), respectively. Excess pentobarbital on the 28th day was administered to sacrifice, and the lungs and lower extremities were harvested.

### 2.8. Histopathological and immunohistochemical studies and gene expression analysis

The size of the tumor was measured as the maximum length of tibia covered by tumoral mass. The differentiation and necrosis in tumor tissue and the number and localization of lung metastases were evaluated in at least six representative sections from each mouse. The degree of tumor necrosis was graded by the ratio of necrotic area to the whole tumor tissue and scored as 1(+) when ≤ 25%, 2(+) when 26%–50%, 3(+) when 51%–75%, and 4(+) when ≥ 75%.

The tissue sections from the tibia and lungs were fixed in 10% formaldehyde and embedded in paraffin blocks. Deparaffinized 4–5 µm sections were mounted on a single slide and stained with hematoxylin and eosin (H+E) for light microscopy examination. For immunohistochemical studies, deparaffinized sections were mounted on poly-l-lysine pretreated membrane slides. The proliferation, differentiation, necrosis, and apoptotic indices of tumor tissue were determined by Ki-67, p27, and caspase-3 immunohistochemical staining. The OS and lung tissues, as well as bronchial epithelial cells of mouse in addition to human tonsil tissue, were used as positive controls in immunohistochemical studies. The mouse periosteal callus tissue from healing femur fracture was used as the positive control for BMP2, and multiple experiments with various dilutions and durations were performed to determine the strongest positivity of osteoblasts against BMP2 antibody for reference in determining the staining intensity scoring. Standard conditions for streptavidin-biotin peroxidase staining were used for immunohistochemical staining. The primary antibodies used in immunohistochemical studies were presented in Table 1. The immunohistochemical evaluations and scoring were performed by a single pathologist. For BMP2 scoring, cytoplasmic staining intensity was assessed, while p27 scoring was assessed separately by the ratio and intensity of positive cells to the whole tumor area and estimation of cumulative staining score (from 1 to 12) by the product of the two scores. For the Ki-67 proliferation index, a total of 1000 cells from the most intense regions under ×400 magnification were counted to estimate the ratio of positive cells. The most intense or wider extent of cytoplasmic staining was assessed for caspase-3 scoring.

**Table 1:** Primary antibodies used in immunohistochemical studies.

Protein	Antibody	Company	Clone/lot	IHC working condition
Bone morphogenetic protein 2	BMP-2	LifeSpan BioSciences	LS-B12549	1:100
Caspase-3	Caspase-3	Atlas Antibodies	A36181	1:200
p27 Kip1	p27	DAKO	M720301	1:50
Ki-67 (MIB 1)	Ki-67 (MIB-1)	Thermo Fisher Scientific	IR626	Ready to use

For the gene expression analysis, the samples were cut in small pieces and homogenized in phosphate buffered saline (PBS, pH 7.4, Thermo Fisher Scientific) by syringe with 26G needle and total RNA was isolated by total RNA isolation kit (Jena Bioscience, Jena, Germany), according to the protocol provided by the manufacturer. Four hundred microliters lysis solution with β-mercaptoethanol was added and vortexed for 15 s. Then, the samples were transferred to filtered tubes and centrifuged in 8000 g for 15 s. The filtered samples were treated with 40 U DNase and incubated at room temperature for 25 min. The samples were twice washed with 500 µL washing buffer provided within the kit and centrifuged in 8000 g for 15 s. Next, the tubes were centrifuged in 13,000 g for 60 s, RNA in the filters were eluted by RNase/DNase free distilled water and centrifuged in 10,000 g for 2 min. Then, filtered tubes were removed, and the remaining samples were placed on ice. The concentration of RNA was measured in 260 nm wavelength using PicoDrop spectrophotometer. Finally, a total of 1 µg isolated RNA was used to synthesize cDNA using Transcriptor High Fidelity cDNA Synthesis Kit (Roche, #5081955001) according to the manufacturer’s protocol.

Real-time PCR using Power SYBR Master Mix (Invitrogen, Applied Biosystems, CA, USA) in LightCycler 480-II (Roche) was performed for studying the expressions of *GFP, BMP2, BMP4, COL1A1, OCN, OPN* and *PF4* genes. The primer sequences were given in Table 2. Relative quantification was determined by second derivative method performed using LightCycler 480 software 1.5 (Roche). Fold expression was calculated by ΔΔC_P_ method with normalization to *beta Actin* ( *ActB* ) housekeeping gene.

**Table 2:** Primer sequences used in RT-PCR.

Protein	Gene	Forward primer	Reverse primer
Bone morphogenic protein 2	BMP2	ACCCGCTGTCTTCTAGTGTTG	TTCTTCGTGATGGAAGCTGAG
Bone morphogenic protein 4	BMP4	ACTTCGAGGCGACACTTCTG	GTCCACCTGCTCCCGAAATA
Collagen type 1, alpha 1	COL1A1	AAGAGGAAGGCCAAGTCGAG	TAAGACAGCTGGGGAGCAAA
Osteocalcin (BGLAP)	OCN	CCTGACTGCATTCTGCCTCT	TCGTCACAATTGGGGTTGAG
Osteopontin (SPP1)	OPN	CCGATGAATCTGATGAGTCCTT	TCCAGCTGACTTGACTCATGG
Platelet factor 4 (CXCL4)	PF4	AGCCCTGAGCTGCTTCTTCT	TCCTGCTTTGATCACCTCCA
Actin, beta	ActB	TTCTACAATGAGCTGCGTGTG	GGGGTGTTGAAGGTCTCAAA

### 2.9. Statistical analysis

The assessment of data distribution was made by visual (histogram and probability tables) and analytic methods (Kolmogorov–Smirnov/Shapiro–Wilk’s test). Median and interquartile range (IQR) were determined according to the variable distributions in definitive analysis. Data were analyzed using the Kruskal–Wallis and Wilcoxon matched-pairs signed-ranks test. The differences among groups were analyzed by the Mann–Whitney U test using Bonferroni correction. SPSS v. 21. for Windows (IBM Corporation, Armonk, NY, USA) was used for all statistical analyses, and a p-value below 0.05 was considered statistically significant. 

## 3. Results

### 3.1. Culture of 143B and hMSCs lines

**Figure 1:**
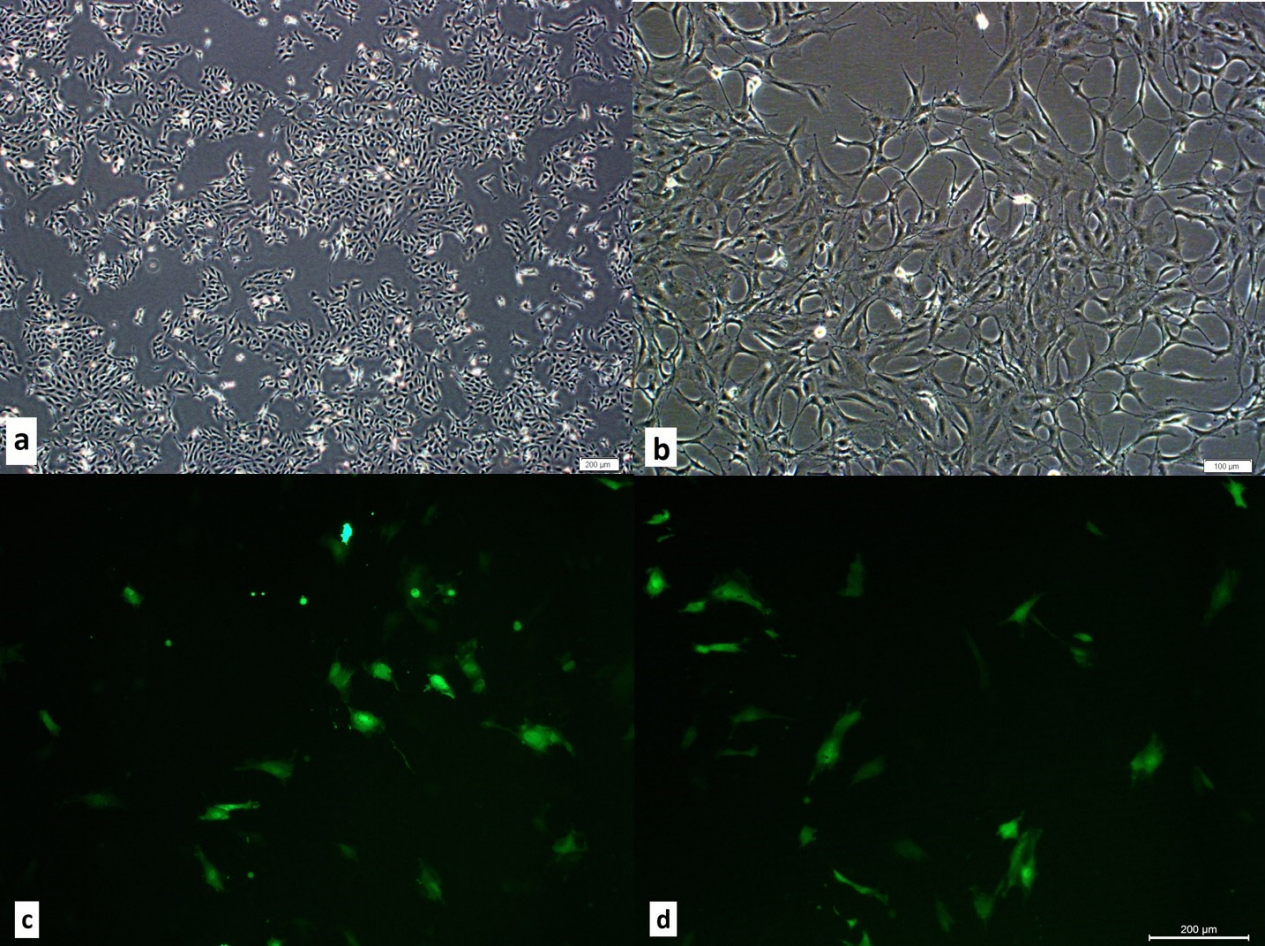
The microphotographs of the cultures of the 143B cells, hMSCs and BMP+hMSCs. a. Morphology of cultured 143B cells. Scale bar 20 μm. b. Morphology of cultured hMSC. Scale bar 20 μm. c. GFP staining of cultured hMSCs in the fluorescent microscopy. d. GFP staining of cultured BMP2+hMSCs in the fluorescent microscopy.

The 143B and hMSCs lines cultured without any contamination, and cells were observed to maintain original morphology during expansion (Figures 1a and 1b).

### 3.2. GFP labeling and BMP2 transfection of hMSCs 

All hMSCs used in this study was labeled with GFP for the localization of the transplanted cells in the tissue. hMSCs were observed directly under fluorescent microscopy at 500–510 nm wavelength. To generate BMP2 expressing mesenchymal stem cells, hMSCs were cotransfected with GFP and BMP2 (BMP2^+^hMSCs) and were observed under fluorescent microscopy after the selective culture for G418 resistance (Figures 1c and 1d). RT-PCR results revealed constitutive BMP2 expression in BMP2^+^hMSCs in the 2ndday of culture.

### 3.3. ELISA measurement of BMP2 levels 

The BMP2 levels in hMSCs and BMP2^+^hMSCs cultures were ≤5.85 pg/mL and 23.4 ± 2 pg/mL, respectively, and the difference between the two cultures was statistically significant (p < 0.01).

**Figure 2:**
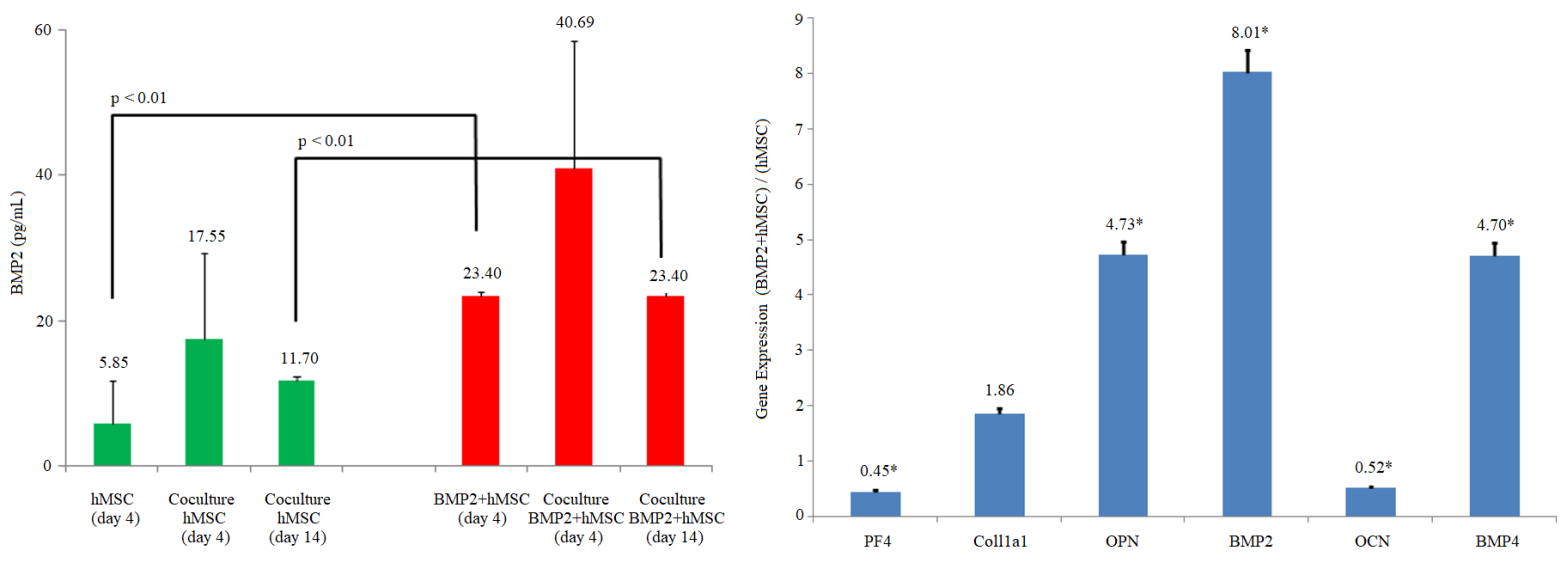
In vitro BMP2 protein levels (pg/mL: picograms per mililiter) and in vitro gene expression analysis. On the left side, the secreted BMP2 level of hMSC and BMP2+hMSC was analyzed before and after the coculture with 143B cells. BMP2 secretion of hMSC and BMP2+hMSC on the 4th day compared and BMP2 secretion increased approximately 4-fold (p < 0.01). BMP2 levels increased in the measurements performed at the end of 4th and 14th days of cocultures (1: 1) with osteosarcoma 143B cell lines. On the right side, comparison of the gene expression of osteogenic differentiation markers in BMP2+hMSC and hMSC in vitro. The expression of PF4, COL1A1, OPN, BMP2, OCN and BMP4 were evaluated. ActB was used as the housekeeping gene in the analysis. The significance of the results with respect to the control group was indicated by asterix (*) when p < 0.01.

The BMP2 level in 143B+hMSCs coculture was 17.55 ± 11.7 and 11.7 ± 0.6 pg/mL in the 4th and 14th days, respectively. The BMP2 level in 143B+BMP2^+^hMSCs coculture was 40.96 ± 17.6 and 23,4 ± 0.6 pg/mL in the 4th and 14th days, respectively, and the difference between the two was statistically significant (p < 0.01) (Figure2). 

### 3.4. In vitro gene expression analysis

The expressions of BMP2, BMP4, COL1A1, and OPN in 143B+BMP2^+^hMSCs coculture compared to 143B+hMSCs coculture was 8-,5-, 2- and 5-fold more, respectively, while OCN and PF4 expressions were 0.5-fold less (Figure2).

### 3.5. In vivo studies

In all mice except one, limping started on the 14th day, and histopathological investigations revealed the presence of tumoral mass around the knee joint, posterior popliteal soft tissue, and proximal tibia. A tumoral embolism in a popliteal lymphatic vessel and a focal lung metastasis was detected in the mouse without limping. Immunohistochemical studies could not be performed in two mice, one from the control and the other from the second group, as the size of their tumors were too small for manipulation. 

**Figure 3:**
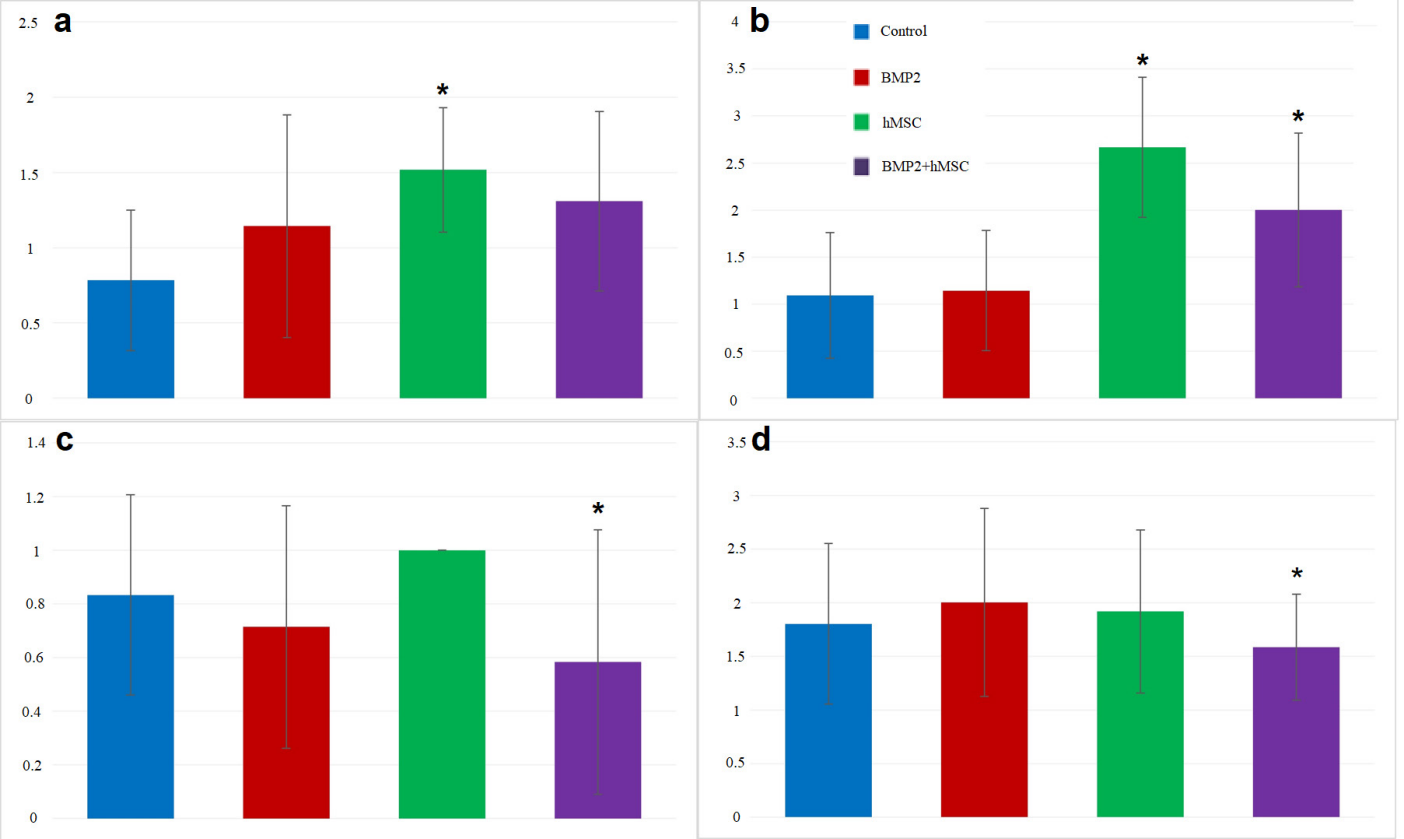
a. The dimensions of tumor (milimeter). The dimensions of tumor in hMSC group were found significantly higher than control group (*p < 0.01). There was no significant relation between the control group and BMP2+hMSC group. b. The extent of necrosis in tumor tissue. The extent of necrosis in the tumor tissue were found significantly greater in hMSC and BMP2+hMSC groups than control group (*p < 0.05). c. The number of metastatic foci. The number of metastatic foci were significantly lower in BMP2+hMSC group than in hMSC group (*p < 0.01). d. p27 immunostaining of tumor tissues. p27 staining level was significantly lower in BMP2+hMSC group than in hMSC group (*p < 0.05).

The tumor diameters ranged between 0.1 and 2.2 cm, while the mean diameter was significantly greater in the hMSCs group compared to controls (p < 0.01) (Figure 3a). The tumoral necrosis was found to be significantly more in the hMSCs group (p < 0.001) and BMP2^+^hMSCs group (p < 0.05) when compared to controls (Figure 3b). Metastasis (bilateral in 20, bilobal in 14) was present in 39 of the mice (multiple in 26). The number of metastatic foci was significantly lower in BMP2^+^hMSCs compared to hMSCs group (p < 0.01) (Figure 3c). 

**Figure 4:**
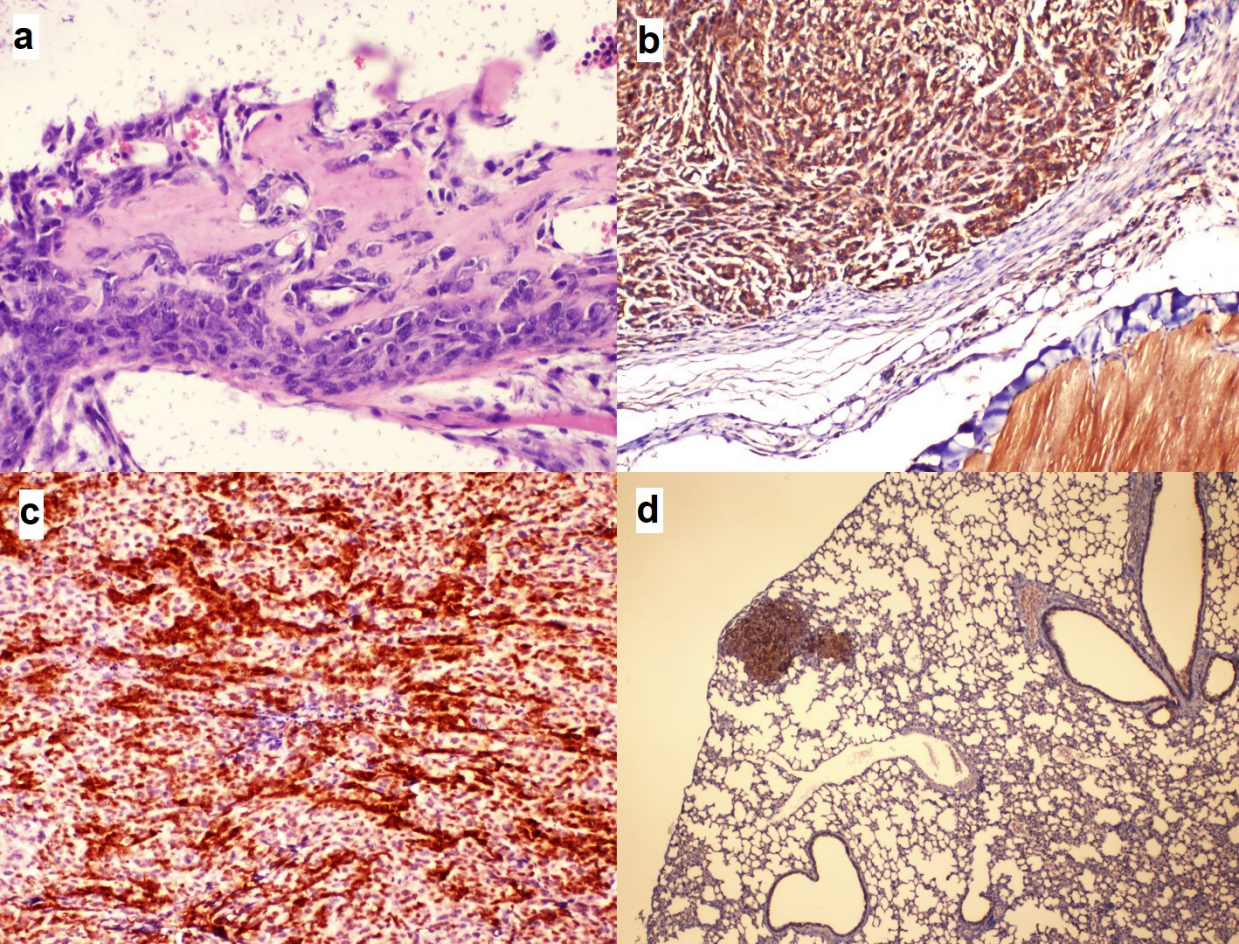
The microphotographs of the morphological and immunohistochemical experiments. a. A microscopic focus showing minimal differentiation of osteosarcoma cells characterized by osteoid matrix production (hematoxylin-eosin staining;  400). b. Osteosarcoma cells displaying diffuse cytoplasmic staining with BMP2 antibody, similar to the striated muscle bundles on the right lower corner of the frame (immunohistochemistry staining;  100). c. Osteosarcoma cells displaying score 12 nuclear and cytoplasmic staining patterns with p27 (immunohistochemistry staining;  100). d. 1 (+) staining intensity similar to bronchial epithelium with caspase-3 displayed in a metastatic focus in lung tissue (Immune histochemistry staining;  40).

The histopathological evaluations in all tumoral tissue showed undifferentiated sarcoma morphology with very high cellularity, extremely atypical spindle cells, extremely high mitotic activity, and necrosis of varying degrees (Figure 4a). Focal differentiation characterized by minimal osteoid production was present in only eight tumors. All tumors were compatible with high-grade fibroblastic type classical osteosarcoma. The immunohistochemical studies revealed positive BMP2 expressions in all osteosarcoma tissue, and no significant difference was observed among groups (p > 0.05) (Figure 4b). 

The expression of p27 in the BMP2^+^hMSCs group was significantly lower than those of the other groups (p > 0.05) (Figures 3d and 4c). We did not observe any significant difference in Ki-67 and caspase-3 expressions among groups (p > 0.05) (Figure 4d).

**Figure 5:**
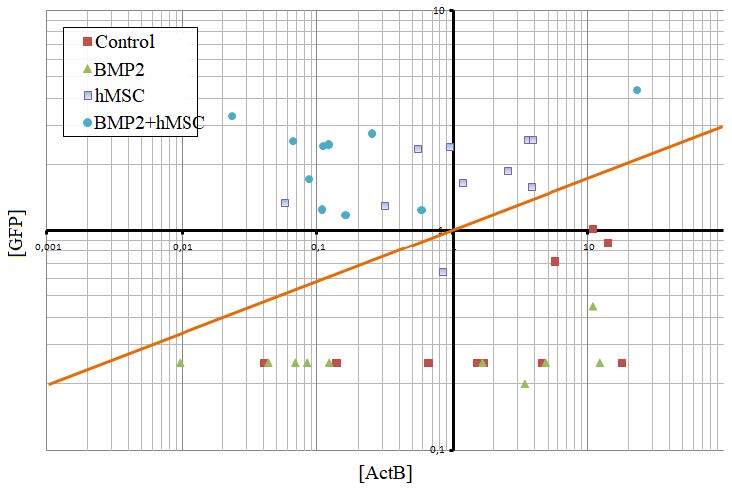
GFP expression in vivo. Screening of GFP expression in the tissue after the transplantation of hMSCs and BMP2+hMSC. The GFP expression could be detected in the samples above the orange line, where only the cell transplanted groups (hMSC and BMP2+hMSC) were localized. No significant GFP expression was measured in the control group and BMP2 groups.

**Figure 6:**
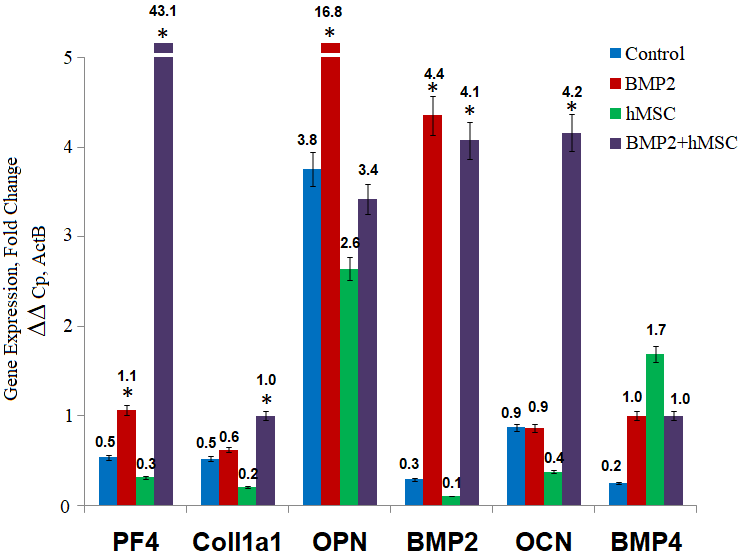
Gene expression analyses in vivo. Gene expression analysis of tissues transplanted with BMP2+hMSC. The expression of PF4, COL1A1, OPN, BMP2, OCN and BMP4 were evaluated. BMP2+hMSC group, in which the transplanted cells were cotransfected with BMP2 and GFP, was compared to BMP2 group, hMSC group and the control group. The significance of the results with respect to the control group was indicated by asterix (*) when p < 0.01.

GFP expressions in hMSCs and BMP2^+^hMSCs groups were significantly more compared to controls and BMP2 group (p < 0.01) (Figure 5). The relative expressions of BMP2, BMP4, OCN, OPN, COL1A1, and PF4 were shown for all groups (Figure 6). In the BMP2 group, there was a significant 10-fold increase in BMP2, a 5-fold increase in both BMP4 and OPN, and a 2-fold increase in PF4 when compared with the controls (p < 0.01). There was a significant 9-fold increase in the relative expression of BMP4 in the hMSCs group when compared to controls (p < 0.01), while the expressions of BMP2, COL1A1, and OCN reduced significantly 3- , 3- , and 2-fold, respectively (p < 0.01). In the BMP2^+^hMSCs group, the BMP2 expression was significantly 10-fold more, while BMP4 was significantly 5-fold more compared to controls. The expressions of OCN, COL1A1, and PF4 in BMP2^+^hMSCs groups compared to controls were significantly increased 4- , 2- , and 80-fold, respectively (p < 0.01).

## 4. Discussion

The target-selective biotherapy has been the subject of recent research. The duration of the biodelivery system activity has been one of the obstacles for efficient cancer biotherapy (Lee et al., 2019). The tumoral site with inflammation and tissue remodeling is the preferential location for MSCs. The pathotropic migratory properties of MSCs make them potential tumor-targeted delivery tools. The critical step for a successful engraftment into the tumor tissue after transplantation of MSCs is the homing (Becker and Riet, 2016). One of the determinants of effective MSC homing is the route of administration. Although intravenous administration is the most commonly used route, a significant amount of hMSCs could be trapped in the lungs as a result of the pulmonary first-pass effect (Khakoo et al., 2006). On the other hand, the intraarterial route due to procedural invasiveness and the embolism risk has not been generally preferred (Qiao et al., 2015). Although not commonly used as the systemic vascular delivery, efficient homing after intraperitoneal administration of hMSCs have been reported previously. Targeted inhibition of OS growth and lung metastasis in mice had been achieved by the intraperitoneal administration of cytosine deaminase/5-fluorocytosine transfected MSCs (NguyenThai et al., 2015). 

The orthotopic xenograft murine OS model was established using 143B OS cell line, known for its aggressive and metastatic properties (Luu et al., 2005). Histopathological studies confirmed the presence of high-grade fibroblastic type classical OS consistent with previous reports (Tian et al., 2019). The GFP expression in hMSCs was significantly higher than that of unlabeled hMSCs confirming successful GFP transfection of hMSCs (p < 0.05). The GFP expressions in hMSCs and BMP2^+^hMSCs were significantly higher than those of the control and BMP2 groups in vivo (p < 0.05). The similar GFP expression levels in cultures and tissues indicated an efficient homing of hMSCs in the targeted OS tissue after intraperitoneal administration and in line with previous studies (NguyenThai et al., 2015).

The MSC behaviors in various tumor microenvironments are distinct. In one study, osteoprotegerin transduced MSCs targeted to mice OS reduced the tumor size (Qiao et al., 2015), while MSCs educated with tumor-secreted extracellular vesicles contributed to OS growth and metastasis in a murine xenograft model in another study (Lagerweij et al., 2018). Recently, discrepant research results on the impact of MSCs on OS have been reported (Zheng et al., 2018). The growth-promoting or metastasis-inducing roles of MSCs were found in some studies (Zheng et al., 2018); the suppression of OS (Gauthaman et al., 2012) and Kaposi sarcoma (Khakoo et al., 2006) were observed in others. BMPs that also regulate cellular expansion and differentiation have been studied for their effects on cancer (Thawani et al., 2010). Despite the findings of higher tumoral expressions, i.e. BMP2 and BMP4 in high-grade OS, their use as a treatment option in OS has been suggested. A recent study demonstrated the promotion of OS cell migration and invasion by BMP2 treatment, while no effect of BMP2 on OS was demonstrated in another study (Tian et al., 2019). However, rapid enzymatic degradation of BMP2 after direct administration led to the search for identifying tools for stable delivery (Tian et al., 2019).

The combined effect of BMP2 and hMSCs had been suggested to be synergistic and more efficient in sustaining and inducing the formation and differentiation of bone tissue (Ishikawa et al., 2007). We observed that the BMP2 levels in the coculture, which initially increased, started to decrease in ten days. The gradual decline in BMP2 levels might indicate that the effect of coculturing has not been steadily sustainable and rather faced the risk of vanishing ultimately. One another possible explanation of the decrease in the BMP2 levels in the 14th day of the coculture might be because of transient transfection of BMP2^+^hMSCs. Nevertheless, the 2-fold more levels on the 14thculture day compared to precoculture values might indicate the possibility of a synergistic effect of BMP2 and hMSCs as reported previously (Ishikawa et al., 2007). Interestingly, an investigation using OS cell lines with high (143B) and low (MNNG-HOS-RFP) metastatic potential demonstrated the enhancement of metastasis by transfer of Ki-ras gene from 143B line in vivo (Tome et al., 2009), giving rise to the question of whether gene transfer between OS cells and MSCs could be a possibility (Zheng et al., 2018). We suspect whether the increase in BMP2 in 143B+hMSCs coculture compared to hMSCs culture might support the possibility of gene transfer or just suggest the interaction of any potential undescribed stimulative factors between the two cell lines. 

The mean tumor size in the hMSCs group was significantly greater than that of the controls (p < 0.01).The extent of the tumoral necrotic area was found to be significantly more in hMSCs (p < 0.001) and BMP2^+^hMSCs (p < 0.05) groups compared to the others. Additionally, the number of metastatic foci was significantly lower in the BMP2^+^hMSCs group compared to the hMSCs group (p < 0.01). The histopathological results indicated that hMSCs treatment caused a greater tumoral size and rapid progression, as shown by more necrotic foci and metastasis; thus, supporting studies that reported a link between tumoral promotion and metastasis in OS and hMSCs (Lagerweij et al., 2018). The BMP2 immunostaining revealed positive reactivity in all study groups, indicating BMP2 gene expression in OS cells. The expression of Ki-67, which was suggested as a prognostic marker due to its association with tumor proliferation and lung metastasis in OS, showed no difference among study groups as expected from the established murine model of OS using 143B cell line, known for its aggressive and metastatic properties (Gallagher et al., 2012). In some studies, BMP2 treatment-related OS growth inhibition was associated with a lower number of Ki-67 positive cells (Xiong et al., 2018). Although statistically not significant, the Ki-67 expression tended to be the lowest in the BMP2 group compared to others, giving possible support to previous results (Xiong et al., 2018). Alternatively, the amount of hMSCs in the tumor environment might be more than the amount that could be compensated by the released amount of BMP2 for a sufficient suppression of the metastatic process. The cytoplasmic expression of the apoptotic protein, caspase-3, was positive in all study groups. Lower levels of caspase-3 were associated with higher grade canine OS, while the administration of canine BMSCs and BMP2 in a murine model of OS resulted in increased expressions of caspase-3 (Reg et al., 2013). When the treated mice compared with the controls, a tendency towards lower expressions of caspase-3 was observed in BMP2 and/or hMSCs groups, suggesting a potential regulatory effect of BMP2 and hMSCs on the balance of cell proliferation/apoptosis cycle favoring apoptosis. We consider that this might be just possible for tumor microenvironment, not for physiological conditions. Recently, the p27 mislocalization by interaction and activation of PAK1-mediated actin polymerization was shown to promote OS metastasis (Chen et al., 2020). Immunohistochemical studies revealed the mislocalization of p27 in tissue sections from all groups; however, the p27 staining in the BMP2^+^hMSCs group was significantly lower than those of others (p < 0.05). Moreover, in vivo evaluations indicated that the number of metastasis in the BMP2^+^hMSCs group was significantly lower than that of the hMSCs group (p < 0.01). The current results could be interpreted as a possible effect of BMP2 transfected hMSCs on the metastatic cascade at tissue level and seemed to be in line with previous results (Chen et al., 2020). 

The interactions among OS and hMSCs were explored by the degree of differentiation of tumoral cells using osteogenic markers like COL1A1,OPN, and OCN that are sequentially expressed matching the temporal pattern of osteoblastic differentiation (Edgar et al., 2007). The failure to reach terminal osteogenic differentiation in OS cells was demonstrated by lower levels of alkaline phosphatase (ALP), Runx2, OSX, and OSP, while the expressions of OPN and OCN were found indifferent to the BMP2 stimulation (Luo et al., 2008). In other studies, the use of modulators like miR148B or coculturing BMP2 transfected MSCs with Endothelial Progenitor Cells were found to cause effective cell proliferation, BMP2 secretion, and better differentiation as demonstrated by higher expressions of COL1A1 and OPN (Lee et al., 2019). In addition to its role in osteogenic differentiation, OPN has also been associated with the metastatic process in OS, and increased expressions were observed in lung metastasis. Recently, the adhesion of OS cells to pulmonary epithelium has been shown to be mediated by OPN (Villanueva et al., 2019). The antiproliferative action of PF4 on MSCs had been demonstrated to be significantly reduced during metastatic progression (Jian et al., 2017). The low expressions of PF4 in OS tissue and high levels in the circulation had been associated with poor outcome.

The in vitro analysis of 143B and BMP2^+^hMSCs coculture demonstrated upregulations of BMP2, BMP4, COL1A1, and OPN expressions. The most and least increases were observed in BMP2 (8-fold) and COL1A1 (2-fold), respectively. On the other hand, OCN and PF4 expressions were reduced in half. These results suggest that osteogenic differentiation had been achieved to an extent; however, not fully completed, as shown by lack of OCN upregulation. Another evidence of incomplete differentiation was the lack of significant cell morphology change and cytoplasmic mineral accumulation in histopathological studies. Thus, the achieved osteogenic differentiation was considered to be at the gene level that was due to the constitutive expression of BMP2. 

The in vivo gene expressions were compared between four groups of mice. As expected, the greatest increase in BMP2 expression (4-fold) was observed in the BMP2 group. On the other hand, the BMP4 expression (9-fold) was the greatest in the hMSCs group. The same amount of increase (5-fold) observed in BMP2 and BMP2^+^hMSCs groups suggested the absence of a significant effect of hMSCs in the presence of BMP2. When we examined the expressions of BMP2 and BMP4 in four groups, a pattern in treated mice groups appeared, indicating an inverse expression level of BMP2 and BMP4 related to the BMP2 treatment in mice. When BMP2 increased in the BMP2+ groups, BMP4 decreased; when BMP4 increased in the hMSCs group, BMP2 decreased. This might be due to the temporal expression of BMP2 and BMP4 in the osteogenic differentiation process, as reported (Edgar et al., 2007; Huang et al., 2007). Additionally, in the absence of BMP2, hMSCs might tend to release BMP4 more, while the presence of BMP2 in the microenvironment might cause increased BMP2 expressions. The increase in COL1A1 expression was slight (0.1-fold) in the BMP2 group, while a 2-fold increase was observed in the BMP2^+^hMSCs group. On the contrary, a 0.5-fold reduction in COL1A1 was found in the hMSCs group. The results of COL1A1 expressions might be interpreted as the targeted efficiency of BMP2 transduced hMSCs had been achieved in the early phases of osteogenic differentiation in OS. The hMSCs might have multimodal actions on differentiation depending on the presence of osteogenic modulators in the tumoral microenvironment. A 4-fold increase in OPN in controls was suggestively due to the metastatic property of the 143B cell line, as higher OPN expressions were recently associated with pulmonary metastasis in OS (Villanueva et al., 2019). In vitro increased expressions of OPN in BMP2^+^hMSCs (5-fold) were found to be decreased (3-fold) in vivo; it can be speculated that the in vitro results reflected the differentiation achieved, while in vivo results reflected the reduction in the metastatic process. The 5-fold increased expression in the BMP2 group compared to 1-fold decreased expression in the hMSCs group further suggested the reduction of the metastatic process by BMP2 and augmentation by hMSCs. The expression of OCN in vitro and in vivo differed greatly with 0.5-fold reduction and 4-fold increase in BMP2^+^hMSCs culture and group, respectively. This difference could be due to the induction of differentiation in vivo by undefined potential modulators, which were absent in the culture conditions. Furthermore, in vivo experiments demonstrated strikingly higher levels of PF4 expression (80-fold) in BMP2^+^hMSCs compared to that of controls, suggesting a significant increase in osteogenic differentiation as well as a reduction in metastatic potential. Considering the gene expression results in general from in vitro and in vivo studies, the expressions of COL1A1 and BMP4 were the ones that demonstrated the least change among other genes. It could be speculated that BMP2 induction might contribute to the differentiation process not in the beginning but at some middle stage as COL1A1 and BMP4 are sequentially expressed earlier than OPN and OCN, respectively. 

Several limitations and strengths of the current study should be acknowledged. In vivo experiments were performed using immunocompromised mice, and it should be noted that the sophisticated network in a normal immune system would possibly interfere at various checkpoints and in malignant processes during OS formation, progression, and metastasis. Secondly, the allowed time for in vitro and in vivo experiments might potentially change the results; it would be rational to expect more advanced differentiation given more time. Third, the BMP2 level in the 143B cell culture did not measured. The comments about the interactions between the 143B cells and hMSCs or BMP2^+^hMSCs might be more precise and accurate if the BMP2 levels in the native 143B cell culture would be known. The conclusions driven from the results of the cocultures of 143B and hMSCs or BMP2^+^hMSCs might just be speculative or exaggerated.

Conclusively, this study, to the best of our knowledge, is the first example of research on the effects of intraperitoneally administered BMP2 transfected hMSCs on human OS. The current results showed that the intraperitoneal route could be efficiently used for targeting hMSCs to the tumoral tissues for an effective gene delivery. In this study, the effects of BMP2 transfected hMSCs on human OS and metastasis were considered to be promising for achieving osteogenic differentiation and reduced lung metastasis.

## Funding

This study was funded by the Scientific and Technological Research Council of Turkey (TÜBİTAK) 1001 research project support program (Project #: 113S264).

## Contribution of authors

Ahmet Sinan Sarı: Conceptualization, Funding acquisition, Formal analysis, Methodology, Investigation, Writing-original draft. Emre Demirçay: Conceptualization, Data curation, Investigation, Writing-review and editing. Ahmet Öztürk: Conceptualization, Data curation, Formal analysis, Writing-review and editing. Ayşen Terzi: Conceptualization, Data curation, Writing-review and editing. Erdal Karaöz: Conceptualization, Formal analysis, Supervision, Writing-review and editing.
